# Frequency of Adverse Events Following Q Fever Immunisation in Young Adults

**DOI:** 10.3390/vaccines6040083

**Published:** 2018-12-13

**Authors:** Emily Sellens, Katrina L. Bosward, Susan Willis, Jane Heller, Rowland Cobbold, Jeannette L. Comeau, Jacqueline M. Norris, Navneet K. Dhand, Nicholas Wood

**Affiliations:** 1 Sydney School of Veterinary Science, Faculty of Science, The University of Sydney, Camperdown, NSW 2006, Australia; katrina.bosward@sydney.edu.au (K.L.B.); jacqui.norris@sydney.edu.au (J.M.N.); navneet.dhand@sydney.edu.au (N.K.D.); 2 University Health Service, The University of Sydney, Camperdown, NSW 2006, Australia; susan.willis@optusnet.com.au; 3 School of Animal and Veterinary Sciences, Charles Sturt University, Wagga Wagga, NSW 2678, Australia; jheller@csu.edu.au; 4 School of Veterinary Science, Faculty of Science, The University of Queensland, Gatton, QLD 4343, Australia; r.cobbold@uq.edu.au; 5 National Centre for Immunisation Research and Surveillance, Westmead, NSW 2145, Australia; j.comeau@dal.ca; 6 Division of Infectious Diseases, Department of Pediatrics, Faculty of Medicine, Dalhousie University, Halifax, NS B3K 6R8, Canada; 7 Discipline of Child and Adolescent Health, Sydney Medical School, The University of Sydney, Camperdown, NSW 2006, Australia

**Keywords:** Q fever vaccination, adverse events, *Coxiella burnetii*

## Abstract

Q fever is a zoonosis of concern in many countries. Vaccination is the most effective means of prevention, and since 1989, Australia has had a licensed Q fever vaccine, Q-VAX^®^. This vaccine was also used in the Netherlands in 2011 following the largest recorded Q fever outbreak globally. There is a paucity of available data regarding adverse events following immunisation (AEFI) for young adult females. Such data are important for informing future vaccination recommendations both within Australia and internationally. This study collected Q fever vaccine (Q-VAX^®^) AEFI data in veterinary and animal science students at Australian universities. Students were enrolled at the time of vaccination and were emailed a link to an online AEFI survey one week later. Of the 60% (499/827) that responded, 85% were female and the median age was 18 years. Local injection site reactions (ISRs) occurred in 98% (95%; CI 96–99%) of respondents, of which 30% (95% CI 24–32%) were severe. Systemic AEFI occurred in 60% (95%; CI 55–64%) of respondents within the seven days following immunisation. Medical attention was sought by 19/499 (3.8%) respondents, of whom one sought treatment at a hospital emergency department. Females were more likely than males to experience any local ISR (odds ratio [OR] 9.3; 95% CI 2.5–33.8; *p* < 0.001), ISRs of greater severity (OR 2.5; 95% CI 1.5–4.2; *p* < 0.001), and any systemic AEFI (OR 1.9; 95% CI 1.1–3.1; *p* = 0.016). These safety data suggest that a high frequency of adverse events following immunisation should be expected in young adults, particularly females. However, the consequences of Q fever disease are potentially far more debilitating.

## 1. Introduction

Q fever is a zoonotic disease in humans causing significant concern for public health in many countries [1,2]. The causative bacterium, *Coxiella burnetii*, is harboured by many domestic and wildlife species and shed into the environment in the placental tissues, urine, milk, and faeces of infected animals [3,4,5]. Transmission to humans is primarily via inhalation of the highly infective and environmentally resilient spore-like phase of the bacterium, which is easily spread over long distances by wind [6,7]. While cattle, sheep, and goats are most commonly implicated in human infections, other species including domestic cats and dogs may also pose a risk [3,8,9,10,11,12].

Acute Q fever is symptomatic in 20–80% of cases, with non-specific clinical signs varying by country, age, and sex [2]. While a flu-like illness is most common, severe symptoms including atypical pneumonia, hepatitis, and myocarditis have been described, and up to 1.5% of acute cases are fatal [1,2]. Patients with co-morbidities are predisposed to persistent focalised *C. burnetii* infection, of which endocarditis in patients with pre-existing heart valve lesions is most commonly reported [1,2]. Infections during pregnancy may be associated with adverse pregnancy outcomes, such as miscarriage and pre-term birth, and predispose the patient to persistent infection [2,13,14,15]. Post Q fever fatigue, a debilitating syndrome presenting as protracted fatigue and often arthralgia and myalgia, occurs in up to 20% of all Q fever cases [16,17,18]. Due to the variable and non-specific presentation of Q fever syndromes, diagnosis and treatment may be delayed or missed in the absence of suspicion and the treatment of persistent infections can be particularly complicated, emphasising the importance of prevention [1].

A whole-cell formalin inactivated Q fever vaccine (Q-VAX^®^; Seqirus, Parkville, Victoria, Australia) has been licensed for use in Australia since 1989. With a reported efficacy of greater than 97% and very few vaccine failures described, vaccination offers the most effective measure for the prevention of Q fever [19,20,21]. In Australia, vaccination is currently recommended for people with a high occupational risk of Q fever, including abattoir workers, farmers, and veterinary personnel [22]. Vaccine uptake is high among occupations where vaccination is mandated, including abattoir workers who are vaccinated prior to commencing their employment, and veterinarians who are vaccinated early in their university study [23,24]. However, uptake is variable among farmers and low among veterinary nurses, the latter reporting concerns about vaccine safety [23,24]. Despite the availability of an effective vaccine, over 500 cases of Q fever are notified annually in Australia [25], and the true burden of disease is likely much greater as many cases may remain undiagnosed; particularly in groups not considered occupationally at-risk.

Internationally, Q-VAX^®^ was used in the Netherlands in 2011 following a large Q fever outbreak. The outbreak occurred from 2007–2009, in which time over 3500 cases of Q fever were notified [26]. In contrast to the occupationally-based use of the vaccine in Australia, a community-based vaccination program was initiated in which patients at high-risk for Q fever complications were identified by general practitioners and referred for Q fever vaccination. Vaccine compliance was high and a total of 1368 patients were vaccinated for Q fever in the Netherlands in 2011 [27].

In the future, there may be a shift towards community-based vaccination within some regions of Australia, as seroprevalence data raise concerns regarding community-based exposure to *C. burnetii*. In Queensland, *C. burnetii* seroprevalence in metropolitan populations (5%) is similar to that of rural/remote populations (5.3%) [28]. In New South Wales (NSW), the Hunter-New England region has an overall seroprevalence of 7%, with some local areas reaching 22%. In this region, seroprevalence among 10–19 year-olds was found to be higher than expected at 5% [29]. Localised outbreaks in Australia also suggest a community-based approach may be required, with cases occurring in people with no history of high-risk exposure activities [30,31], and an outbreak at an abattoir in South-Western Sydney, New South Wales, raising concerns due to its close proximity to residential properties and a school [32].

Sufficient and available vaccination safety data are required to support an increase in vaccine uptake among occupational cohorts where vaccination is not mandated, and to support any future recommendations for a change from an occupationally-based to a community-wide vaccination program within Australia. Pre-licensure adverse events following immunisation (AEFI) data for Q-VAX^®^ were collected from 1981–1988 during trials in Australian abattoirs where vaccinees were predominantly male (mean age 29 years) [33,34,35]. More recently, AEFI data were collected during the large scale vaccination program in the Netherlands [36]. Although this study included a greater proportion of females (40%) than the Australian studies, vaccinees were older (median 67 years) and suffered significant co-morbidities [36].

Additional data are available from the Australian Government Therapeutic Goods Administration National Database of Adverse Event Notifications (DAEN), a passive surveillance system. This system provides important safety data, particularly regarding serious AEFI for which reporting is mandatory. Serious AEFI are defined as such where death, life-threatening illness, hospitalisation, persistent or significant disability/incapacity, or a congenital anomaly/birth defect occurs [37]. Generally though, the surveillance system underestimates overall AEFI [38], as demonstrated during Australia’s national Q fever management program where only 86 AEFI (eight of which were serious) were reported during the program (2001–2004) despite the administration of close to 50,000 vaccines [23]. It is also not possible to determine the AEFI reporting rate from this database, as the number of Q-VAX^®^ doses administered over time is not reliably recorded.

Consequently, there is a paucity of detailed AEFI data available for young adults, particularly females, and a complete lack of data for paediatric populations (<16 years old). This study aims to provide safety data for Q-VAX^®^ in young adults in a format that is more descriptive than previously reported, with a focus on young adult females in particular. These data will provide useful information for both medical practitioners and young adult vaccinees, and for any future consideration of vaccine trials in paediatric populations.

## 2. Materials and Methods

### 2.1. Subjects

University students enrolled in animal science or veterinary science courses in Australia are routinely vaccinated against Q fever in their first year of study, following routine pre-vaccination testing procedures. University students are mostly young adults (<25 years old) and in Australia over 75% of the veterinary science cohort are women. Students from three universities in Australia were enrolled in the study at the time of their Q fever vaccination: (1) veterinary science students at The University of Sydney in 2013 and 2014, (2) veterinary science, animal science, equine science, and veterinary technology students at Charles Sturt University in 2014, 2015 and 2016, and (3) veterinary science and veterinary technology students from The University of Queensland in 2014 and 2015. Students who were negative on pre-vaccination screening and subsequently received the Q fever vaccination were invited to participate in the study.

Primary ethics approval was granted by the University of Sydney human research ethics committee (Protocol #15012; Project # 2012/1686). Secondary approvals were granted by Charles Sturt University School of Animal and Veterinary Sciences ethics in human research committee (protocol #2015/003), and the University of Queensland institutional human research ethics committee (approval #2014000328).

### 2.2. Q-VAX^®^ and Q-VAX^®^ Skin Test

Q-VAX^®^ and Q-VAX Skin Test^®^ contain whole cell formalin-inactivated Phase 1 *Coxiella burnetii* Henzerling strain. The Q-VAX^®^ vaccine contains a minimum of 25 µg of antigen in 0.5 mL of aqueous solution, which is administered subcutaneously in the upper arm [39]. The Q-VAX^®^ Skin Test contains 2.5 µg antigen per 0.5 mL of aqueous solution and is further diluted in sodium chloride prior to administration. The final 0.1 mL dose contains 16.7 ng of antigen and is delivered intra-dermally into the volar surface of the mid-forearm as part of the pre-vaccination screening process [39].

In addition to antigen, Q-VAX^®^ and Q-VAX Skin Test^®^ contain sodium chloride, sodium phosphate-monohydrate, and sodium phosphate-dihydrate. Thiomersal 0.01% *w/v* is added as a preservative [39].

### 2.3. Pre-Vaccination Testing

Prior to vaccination, as part of the recommended protocol prescribed by the vaccine manufacturer [39], participants were questioned by a medical practitioner regarding the possibility of previous exposure to *C. burnetii* and underwent serological and intra-dermal skin testing to assess for pre-existing sensitisation to *C. burnetii* antigens resulting from prior natural exposure or vaccination. Blood was collected and the intra-dermal skin test injection (Q-VAX Skin Test^®^) given on the same day. The skin test reaction was subjectively assessed by a medical practitioner, experienced in reading Q VAX^®^ skin tests, seven days post-injection. Blood samples were sent to commercial labs for serological profiling: (1) University of Sydney samples to Douglass Hanley Moir (Macquarie Park, NSW, Australia) utilising an indirect immunofluorescence assay (IFA), (2) University of Queensland samples to the Queensland Medical Laboratory (Murarrie, QLD, Australia) utilising an enzyme-linked immunosorbent assay (ELISA), and (3) Charles Sturt University samples to Symbion Laverty Pathology (Macquarie Park, NSW, Australia) utilising an ELISA. Vaccination was administered if both the serology and skin test were negative, and no history of probable prior exposure was identified.

### 2.4. Data Collection and Survey Design

Eligible patients were enrolled by university medical or research staff immediately following their vaccination. Consent forms were signed in which participants provided a contact email address. One week after vaccination, vaccinees enrolled in the study were emailed a link to participate in an online survey administered via the Survey Monkey platform. The survey contained two closed and seven semi-closed questions pertaining to local and systemic adverse events following immunisation. Within this survey, participants were asked if they had experienced each local reaction, the size of each reaction, and at what time point following vaccination the local reactions had occurred. For systemic events, participants were asked if they had experienced each event within the seven days following vaccination. Sex was also asked as a closed question in the survey, while age and vaccination location were recorded at enrolment and later matched with questionnaire responses.

### 2.5. Statistical Analysis

Descriptive statistics were generated for demographic and adverse events data to assess the frequency and severity of AEFI reported. The frequency of AEFI reported was compared between females and males using generalised linear mixed modelling (PROC GLIMMIX procedure) in the SAS© statistical program (SAS Institute Inc., Cary, NC, USA). Outcome variables for the frequency of AEFI were binary, reflecting whether each AEFI was reported to have occurred or not. Sex was tested as a fixed effect for each outcome. A *p*-value of <0.05 was considered statistically significant. The odds ratios and their 95% confidence limits were presented. The year of vaccination and the location of vaccination were included as random effects to account for clustering.

Local injection site reactions (ISRs) were further graded for severity based on the level of pain reported and measurement of the area of erythema and/or swelling. The criteria for grading local ISRs, outlined in Table 1, were the same as those used by Schoffelen et al. [36] (2014) for Q-VAX^®^. The overall grade for ISRs assigned for each vaccinee corresponded with the highest grade reported for any of the individual local ISRs. The ordinal outcome variable “ISR grade” was comprised of three categories; grade 1 (mild), grade 2 (moderate), and grade 3–4 (severe). This outcome was compared between females and males, also using generalised linear mixed modelling with year and location of vaccination included as random effects. 

## 3. Results

### 3.1. Demographics

A total of 839 vaccinees were enrolled at the time of vaccination and provided consent and contact details for participation in the online survey. Of these, 12 were not contactable due to incorrect email addresses. Survey responses were received from 499 vaccinees across the three locations from 2013–2016, resulting in a response rate of 60% (499/827). The majority (85%) of respondents were female, and the median age was 18 years (interquartile range 2 years). Age distribution was similar for both females and males (Table 2). The demographics of respondents were much the same across the three university locations (Table 2).

### 3.2. Injection Site Reactions

**Figure 1:**
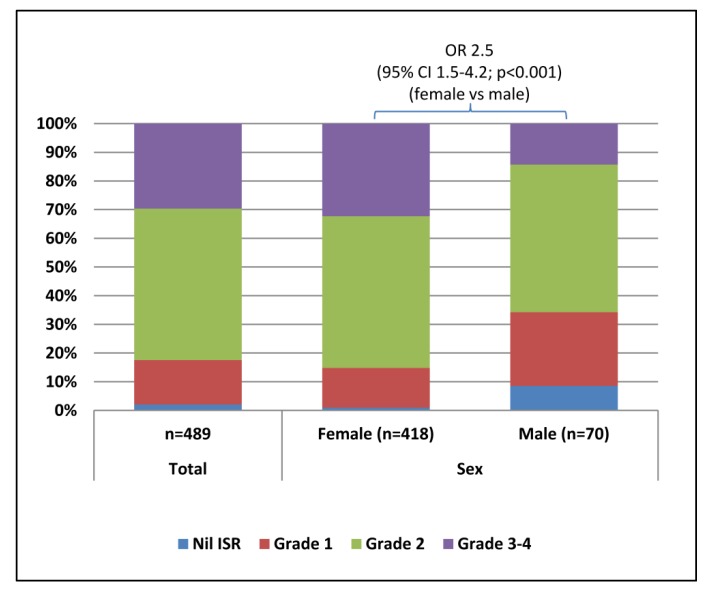
Proportion of total respondents and of females and males assigned to each grade of injection site reaction (ISR) following Q fever immunsation. Females were more likely to report severe ISRs.

Local ISRs were reported by 98% (95% CI 96–99%) of respondents. Injection site pain occurred in 95% (95% CI 92–96%) of respondents, while erythema and swelling were less common, each reported by 58% (95% CI 54–62%) of respondents (Table 3). Pronounced ISRs (grades 3–4) occurred in 30% (130/473; 95% CI 24–32%) of respondents (Figure 1). The majority (76%; 366/481) of ISRs appeared within 24 hours of vaccination, 23.5% (113/481) between days 2–5, and less than one percent (2/481) occurred more than five days post-vaccination. Females were significantly more likely to report local ISRs (Table 3) and ISRs of a higher grade (Figure 1) than males. 

### 3.3. Systemic Adverse Events

Systemic AEFI occurred in 60% (95% CI 55–64%) of respondents within the seven days following immunisation. Headache (44%; 95% CI 40–48%) and lethargy (43%; 95% CI 38–47%) were most commonly reported. Joint pain was experienced by 25% (95%; CI 21–29%) and fever was reported by 17.2% (95%; CI 14–21%) of respondents (Table 4). Females were significantly more likely to report experiencing any systemic vaccine reaction, and more specifically, lethargy (Table 4).

### 3.4. Medical Attention Following Vaccination

Medical attention for AEFI was sought from a health provider by 19/499 (3.8%) respondents. Of these, nine sought attention for both local ISRs and systemic AEFI, six sought attention for only local ISRs, and four sought attention for only systemic AEFI. These vaccinees sought help from general practitioners (*n* = 7), University Health Services (*n* = 7), and pharmacists (*n* = 3). One sought help from a doctor within their family, and one (0.2%) presented to an emergency department experiencing a pronounced (grade 4) injection site reaction and all four systemic events. Data on the outcome of this last patient were not available through the survey.

## 4. Discussion

This study successfully recruited a large number of young adults to provide detailed data for adverse events following Q fever immunisation with Q-VAX^®^. Further supporting data are provided regarding the safety of this vaccine in this age bracket, with an emphasis on young adult females who have been under-represented in previous reports. The overall proportion of respondents in this study that experienced local and/or systemic adverse events exceeded pre-licensure clinical trial data reported for Q-VAX^®^ (Table 5). Schoffelen et al. [36] (2014) similarly reported a higher proportion of AEFI following Q-VAX^®^ (Table 5). These increased AEFI may be explained by demographic, social and educational factors, and methodology.

Both this study and Schoffelen et al. [36] (2014) identified that a greater proportion of females reported local and systemic AEFI, and AEFI of a higher severity, following Q fever vaccination compared to males. Gidding et al. [23] (2009) also identified a 2:1 ratio of females to males in passive adverse event notifications to the DAEN surveillance system during Australia’s national Q fever vaccination program from 2001–2004 [23]. These findings provide evidence that females are more likely to experience AEFI than males and explains why the overall proportion of vaccinees experiencing AEFI in this cohort of predominantly female vaccinees was higher than pre-licensure clinical trials, where the majority of vaccinees were male. Females also experienced more severe AEFI symptoms in these studies, with the exception of Gidding et al. [23] (2009) where males reported more severe symptoms. The latter finding was drawn from passive surveillance and may reflect a decreased propensity for males to report less severe AEFI than females.

Sex based differences in vaccine reactogenicity are reported for many vaccines with predominantly females reporting increased AEFI [40,41]. The cause of this difference is multifactorial and hypotheses include (1) elevated humoral and cell-mediated immune responses in females in response to vaccination, (2) increased perception of pain in females, (3) increased likelihood of subcutaneous rather than intramuscular deposition of vaccines in females due to a thicker subcutaneous layer than males, and (4) the social expectations of males to be stoic and pain tolerant [40,41,42,43].

The immune response to *C. burnetii* is strongly influenced by sex, with the majority of genes modulated following infection being sex-dependent [44]. Cell mediated immunity (CMI) plays an essential role in the control of early *C. burnetii* infection [45]. Compared to males, females exhibit stronger CMI responses and have reduced bacterial numbers following *C. burnetii* infection [46]. However, CMI is associated with more severe ISRs following immunisation [40,43], which explains why females exhibited increased ISRs of greater severity following Q fever vaccination. While the proportion experiencing severe ISRs (30%) may have been exaggerated due to self-reporting from recall, the result is comparable to that observed by Schoffelen et al. [36] (2014) who reported these reactions in 20% of all respondents from a cohort comprised of older patients with significant comorbidities, including immunosuppression. Sex differences in immune response also contribute to males more often exhibiting clinical Q fever disease of greater severity following natural infection [44].

Age also influences immune responses, with younger age appearing protective against clinical Q fever disease following *C. burnetii* infection [3]. This can be explained by a decline in T cell function with increasing age [47]. As T cells are essential for *C. burnetii* clearance [45], a more robust response in younger people provides immune protection from disease, but may also increase the likelihood and severity of AEFI following vaccination. Schoffelen et al. [36] (2014) reported increased AEFI in the younger (<50 years) non-immune suppressed cohort of vaccinees; exceeding 90% for females in this category [36]. The cohort in this study consisted of young adults, and age may have contributed to the increased AEFI reported. However, the effect of sex appears to be more important as pre-clinical vaccine trials also included younger workers.

Educational and social factors may have contributed to the increase in AEFI reported here compared to pre-licensure clinical trials. The vaccinees in this study were university students with an interest in science and medicine. They were likely more educated than the vaccinees in the pre-licensure clinical trials, most of whom were abattoir workers [33], and may have been more highly motivated to observe and report even minor adverse events, contributing to the increase in AEFI reported here. Indeed, medical students participating in vaccine studies have similarly demonstrated increased reporting of AEFI [41]. Additionally, young adults are more likely to experience health anxiety, which can lead to exaggeration of symptoms [48]. This may have contributed to the number of respondents seeking medical attention for their vaccine reactions. Young adults demonstrated increased healthcare seeking behaviours for AEFI following the tetanus and diphtheria-toxoid vaccine [49], and such behaviour may have been further exaggerated for this cohort if they had recently moved away from home and support networks to commence their studies.

The main limitation of this study was the response rate of 60%, which may have favoured participation from vaccinees that experienced an AEFI, and from females generally as they are known to be more likely to participate in surveys [40,42]. Indeed, Schoffelen et al. [36] (2014) reported a response rate of 71% (74% for females; 68% for males) and identified that 80% of the respondents had experienced local AEFI, compared to only 31% of vaccinees who did not respond but were questioned at a later follow-up. Thus, the overall frequency of AEFI may be inflated by an over-representation of symptomatic respondents and under-representation of males generally, who would have been more likely to be asymptomatic. However, the difference between females and males may be even more pronounced than that reported due to under-representation of males. As the sex of vaccinees was not recorded at the time of enrolment in this study, and AEFI data for non-responders is not available, it is not possible to assess the extent of gender bias in these results. Some bias is expected, as 85% of respondents were female compared to 79% of veterinary students commencing their studies between 2013 and 2016 in Australian universities [50]. Consequently, these results represent a worst-case scenario for the cohort studied. The best-case scenario reflects a frequency of 59% (489/827) for injection site reactions and 36% (297/827) for systemic reaction. This assumes all non-responders were asymptomatic, which is unlikely, and still results in a higher reported frequency of AEFI than pre-licensure trials.

Information collected from vaccinees was limited to one week following vaccination. A serious AEFI will not have been captured in this study if it occurred more than one week following vaccination or resulted in the vaccinee being too ill to participate in the survey. One vaccinee did report seeking medical attention at a hospital emergency department, which may have been classified as serious if hospitalisation was required; however, the outcome of this patient was not captured in this survey. A search of the DAEN revealed 33 case reports of adverse events, none of which recorded death as an outcome following administration of Q-VAX^®^ from January 2013–June 2016; the time frame in which this study was undertaken [51].

## 5. Conclusions

These data contribute useful information on the safety profile of Q-VAX^®^ in young adults, with an emphasis on females who have been under-represented in previous studies and for whom detailed AEFI data has not been specifically reported. Q-VAX^®^ was found to be reactogenic among respondents, and a high frequency of vaccine reactions should be expected in young adults, particularly females. However, AEFI were mostly non-severe and few vaccinees sought medical attention. Ideally, a less reactogenic but equally effective Q fever vaccination is needed. Until such a vaccine is available, the high likelihood of experiencing transient non-severe adverse events following Q fever immunisation should not deter people from seeking vaccination, as the consequences of Q fever disease are potentially far more debilitating. These results are important for policymakers and healthcare providers as they provide further safety data on young adults and females and would be useful if a trial of this vaccine in younger adolescents and children was to be considered in the future.

## Figures and Tables

**Table 1:** Criteria for grading the severity of local injection site reactions (ISRs) following Q fever vaccination. Vaccinees were assigned an overall ISR grade corresponding to the highest reported grade across the three symptoms.

Grade	Severity	Description of Pain	Area of Erythema or Swelling
1	Mild	Pain to the touch, no obstruction of use	<2.5cm
2	Moderate	Pain on movement, some interference with normal activity	2.5 to <7.5cm
3	Severe	Considerable pain in rest, obstruction of use	7.5 to <15 cm
4	Extensive	-	15 cm or greater

**Table 2:** Demographic information of Q fever vaccinees participating in the adverse events following immunisation study across the three study locations.

Variable	University of Sydney	University of Queensland	Charles Sturt University	Total Vaccinees
**Sex**				
Females *n* (%)	194 (85.5)	106 (80.9)	124 (87.9)	424 (85.0)
Males *n* (%)	33 (14.5)	25 (19.1)	16 (11.3)	74 (14.8)
Unspecified *n* (%)	0 (0)	0 (0)	1 (<1)	1 (<1)
Column Total	227 (100)	131 (100)	141 (100)	499 (100)
**Age**				
Range (years)	17–43	17–29	17–38	17–43
Mean (years)	20.3	19.0	19.7	19.8
Median (years)	19.0	18.0	19.0	18.0
Interquartile Range	3.0	3.0	2.0	2.0
**Year of vaccination**				
2013 *n* (%)	143 (63.0)	0 (0)	0 (0)	143 (28.7)
2014 *n* (%)	75 (33.0)	21 (16.0)	19 (13.5)	115 (23.0)
2015 *n* (%)	9 (4.0)	110 (84.0)	55 (39.0)	174 (34.9)
2016 *n* (%)	0 (0)	0 (0)	67 (47.5)	67 (13.4)
Column Total	227 (100)	131 (100)	141 (100)	499 (100)

**Table 3:** Local injection site reactions (ISRs) reported by respondents following Q fever vaccination. The odds ratio for reporting “yes” for each ISR is shown for females versus males.

All Respondents		Sex	
		Females	Males
Any Local Injection Site Reaction			
Yes *n* (%)	489 (98.0)	420 (99.1)	68 (91.9)
No *n* (%)	10 (2.0)	4 (<1)	6 (8.1)
Column Total *n* (%)	499 (100)	424 (100)	74 (100)
OR (95% CI; *p*-value)	-	9.3 (2.5–33.8; <0.001)	ref
Injection Site Pain			
Yes *n* (%)	473 (94.8)	411 (96.9)	61 (82.4)
No *n* (%)	26 (5.2)	13 (3.1)	13 (17.6)
Column Total *n* (%)	499 (100)	424 (100)	74 (100)
OR (95% CI; *p*-value)	-	6.7 (3.0–15.2; <0.001)	ref
Injection Site Swelling			
Yes *n* (%)	289 (57.9)	257 (60.6)	32 (43.2)
No *n* (%)	208 (41.7)	165 (38.9)	42 (56.8)
Not Specified *n* (%)	2 (<1)	2 (0.5)	0 (0)
Column Total *n* (%)	499 (100)	424 (100)	74 (100)
OR (95% CI; *p*-value)	-	2.5 (1.5–4.3; <0.001)	ref
Injection Site Erythema			
Yes *n* (%)	289 (57.9)	257 (60.6)	31 (41.9)
No *n* (%)	207 (41.5)	165 (38.9)	42 (56.8)
Not Specified *n* (%)	3 (<1)	2 (<1)	1 (1.4)
Column Total *n* (%)	499 (100)	424 (100)	74 (100)
OR (95% CI; *p*-value)	-	3.3 (1.9–5.7; <0.001)	ref

OR; Odds Ratio. CI; Confidence interval. Ref; reference category for odds ratio. Odds ratio adjusted for year and location of vaccination.

**Table 4:** Systemic adverse events experienced by Q fever vaccinees. The odds ratio for reporting “yes” for each adverse event is shown for females versus males.

All Respondents		Sex	
		Females	Males
Any Systemic AEFI			
Yes *n* (%)	297 (59.5)	263 (62.0)	34 (45.9)
No *n* (%)	202 (40.5)	161 (38.0)	40 (54.1)
Column Total *n* (%)	499 (100)	424 (100)	74 (100)
OR (95% CI; *p*-value)	-	1.9 (1.1–3.1; 0.016)	ref
Fever			
Yes *n* (%)	86 (17.2)	76 (17.9)	10 (13.5)
No *n* (%)	409 (82.0)	344 (81.1)	64 (86.5)
Not Specified *n* (%)	4 (<1)	4 (<1)	0 (0)
Column Total *n* (%)	499 (100)	424 (100)	74 (100)
OR (95% CI; *p*-value)	-	1.4 (0.7–2.8; 0.384)	ref
Headache			
Yes *n* (%)	219 (43.9)	194 (45.8)	25 (33.8)
No *n* (%)	276 (55.3)	226 (53.3)	49 (66.2)
Not Specified *n* (%)	4 (<1)	4 (<1)	0 (0)
Column Total *n* (%)	499 (100)	424 (100)	74 (100)
OR (95% CI; *p*-value)	-	1.6 (1.0–2.8; 0.058)	ref
Lethargy			
Yes *n* (%)	213 (42.7)	190 (44.8)	23 (31.1)
No *n* (%)	283 (56.7)	233 (55.0)	49 (66.2)
Not Specified *n* (%)	3 (<1)	1 (<1)	2 (2.7)
Column Total *n* (%)	499 (100)	424 (100)	74 (100)
OR (95% CI; *p*-value)	-	1.7 (1.0–3.0; 0.048)	ref
Joint Pain			
Yes *n* (%)	123 (24.6)	112 (26.4)	11 (14.9)
No *n* (%)	374 (74.9)	311 (73.3)	62 (83.8)
Not Specified *n* (%)	2 (<1)	1 (<1)	1 (1.4)
Column Total *n* (%)	499 (100)	424 (100)	74 (100)
OR (95% CI; *p*-value)	-	2.0 (1.0–3.9; 0.055)	ref

OR; Odds Ratio. CI; Confidence interval. Ref; reference category for odds ratio. Odds ratio adjusted for year and location of vaccination.

**Table 5:** Summary of the proportion of overall respondents experiencing acute adverse events following Q fever vaccination in the current study and other available published data for Q-vax^®^.

	Independent Data		Clinical Trial Data	Registration Holder Surveillance Data
	Current Study	Schoffelen et al. [36] (2014)	Marmion et al. [33] (1990)	Seqirus [39] (2017)
Study population	Veterinary students; median age 18 years, predominantly female	Persons in community with high risk of Q fever due to comorbidities; median age 67 years	Abattoir workers; median age 29 years, predominantly male	Not applicable
Number of vaccinees for which AEFI results are reported	499	970	464	Not applicable
Any local or systemic AEFI	98%	82%	*	*
Any Local ISR	98%	80%	*	*
Injection Site Pain	95%	*	48%	≥10%
Injection Site Swelling	58%	*	*	≥10%
Injection Site Erythema	58%	*	33%	≥10%
Any Systemic AEFI	60%	43%	*	*
Headache	44%	*	9%	<10% and ≥1%
Lethargy	43%	*	*	<1% and ≥0.1%
Joint Pain	25%	*	*	<0.01%
Fever	17%	9%	0.2%	<1% and ≥0.1%

* Data not published. ISR; Injection site reaction; AEFI; adverse event following immunisation.
